# Supplementary figures and images for: Inflammatory stress signaling via NF-kB alters accessible cholesterol to upregulate SREBP2 transcriptional activity in endothelial cells
Source: eLife. 2022 Aug 12;11:e79529. doi: 10.7554/eLife.79529 (PMC9395194; doi:10.7554/eLife.79529)

a.

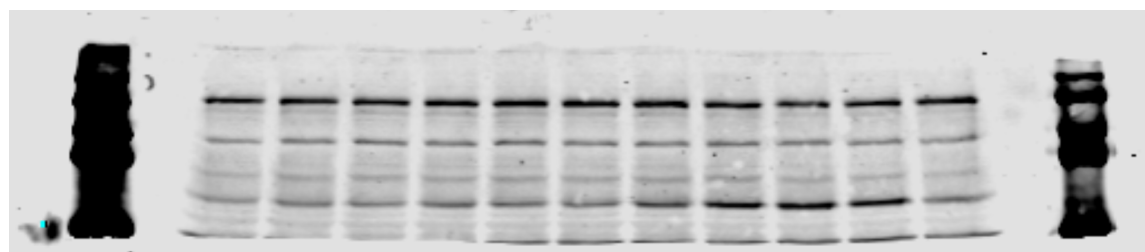

-P  
SREBP2  
-C

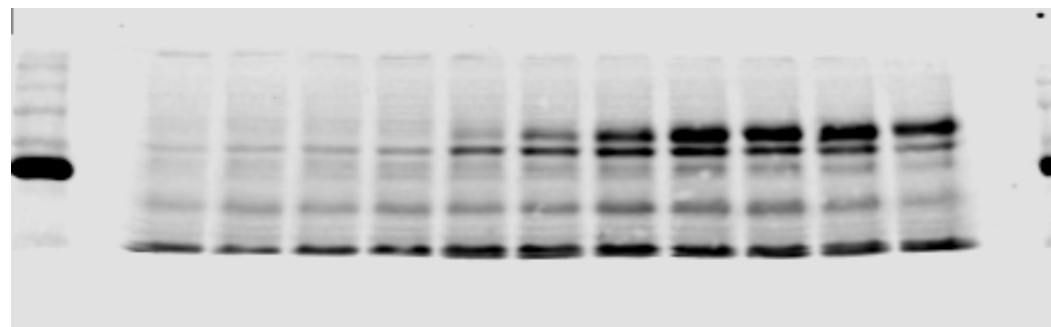

ICAM1

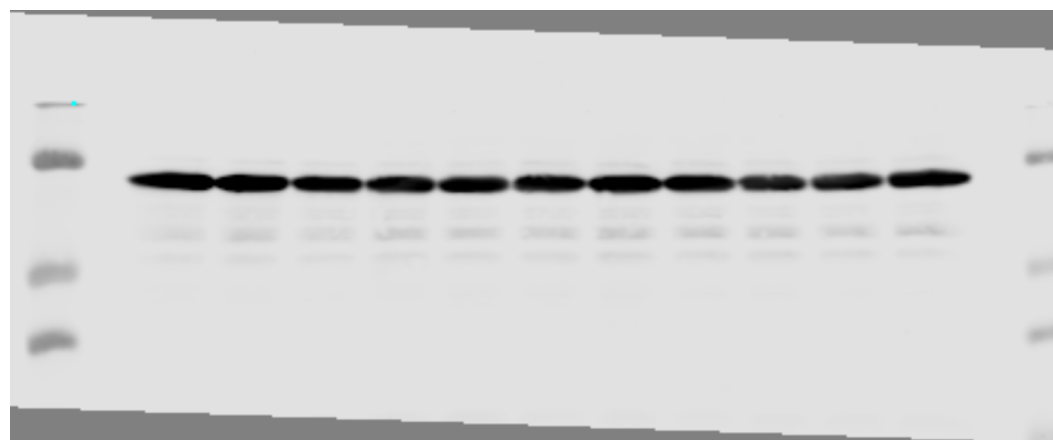

GAPDH

c.

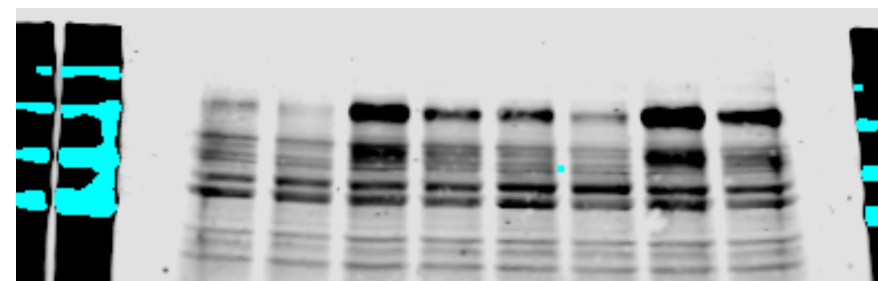

LDLR

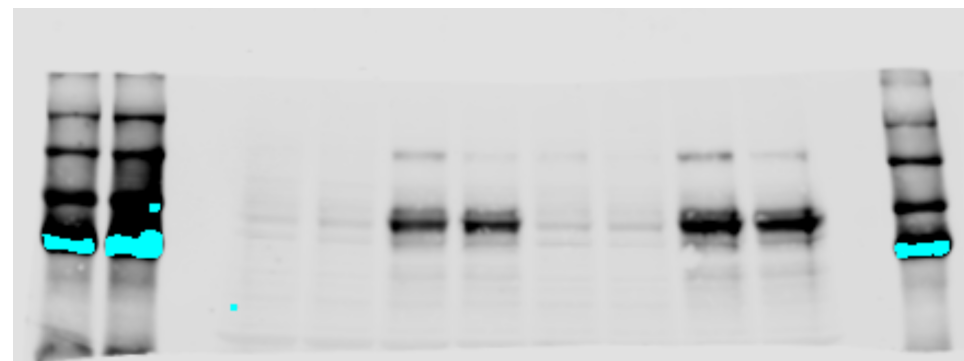

ICAM1

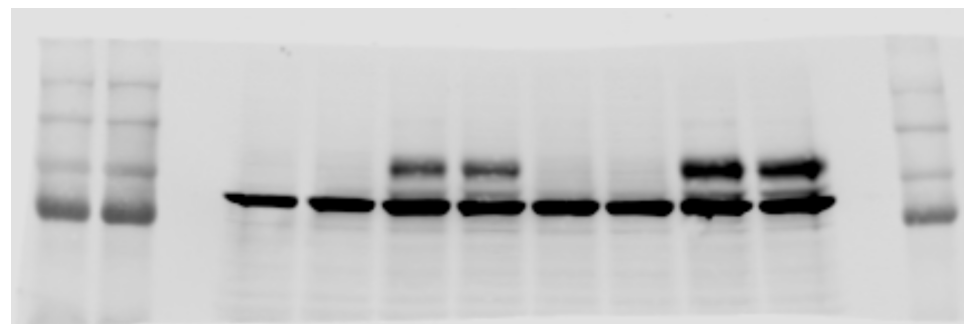

VCAM1

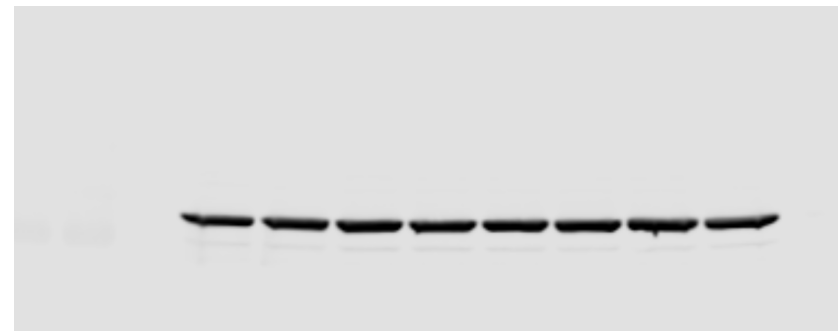

HSP90

Supplement: Figure 2—source data 1. [file elife-79529-fig2-data1.pdf]

**a.**

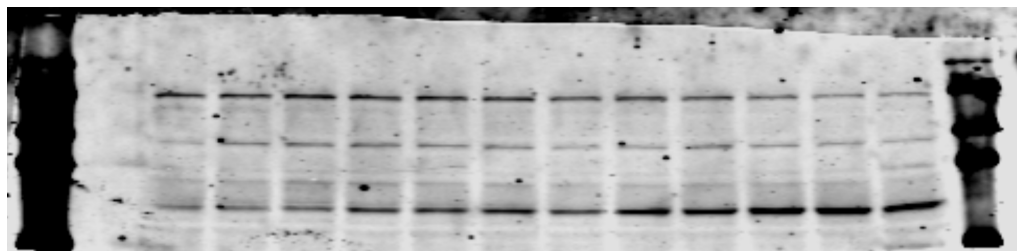

-P  
SREBP2  
-C

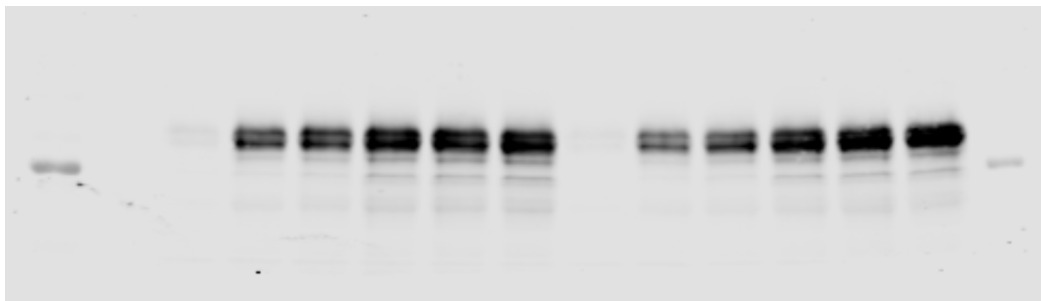

VCAM1

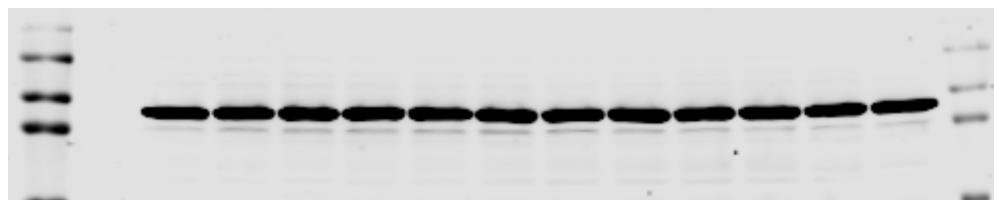

HSP90

**c.**

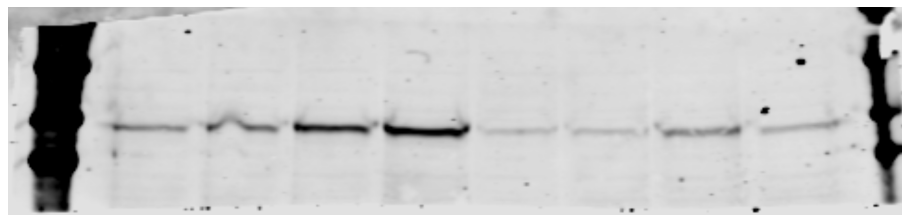

HMGCR

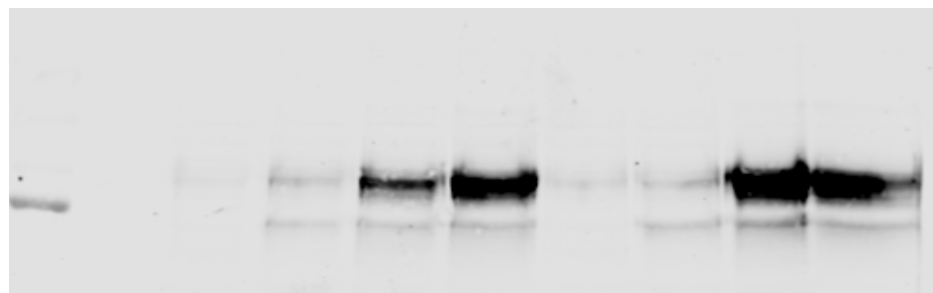

ICAM1

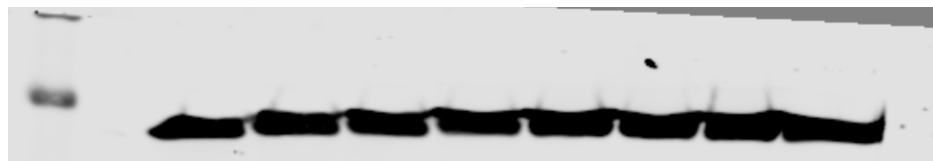

GAPDH

Supplement: Figure 2—figure supplement 1—source data 1. [file elife-79529-fig2-figsupp1-data1.pdf]

**b.**

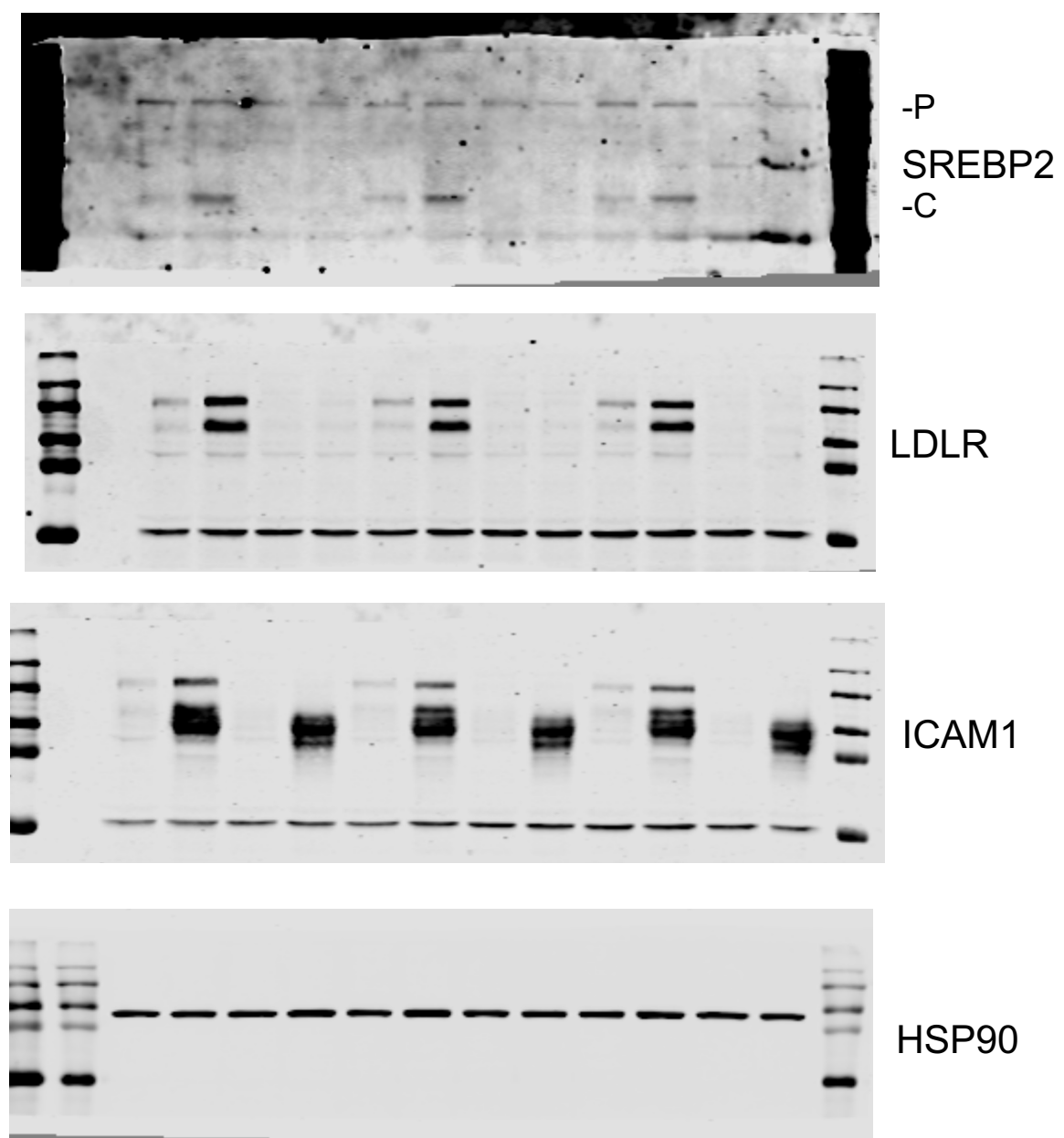

**c.**

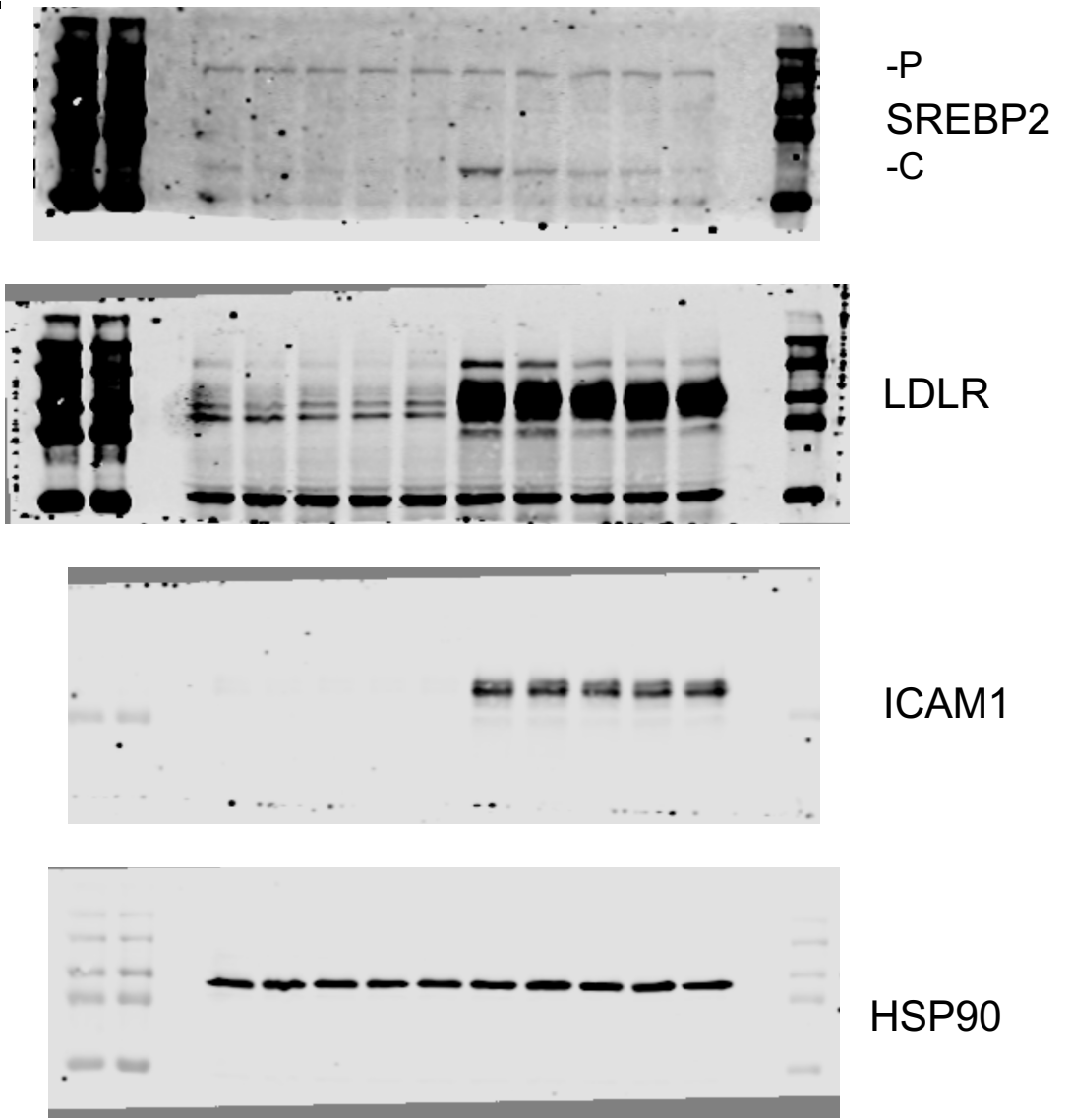

d.

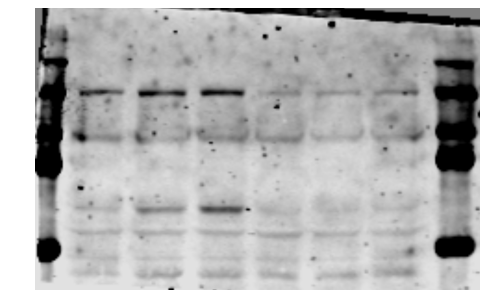

-P  
SREBP2  
-C

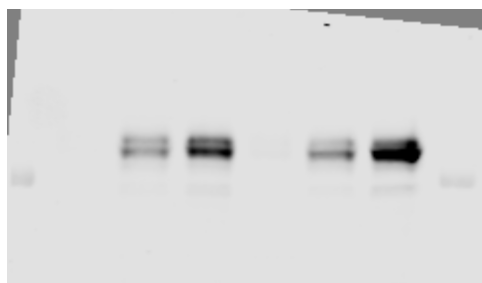

ICAM1

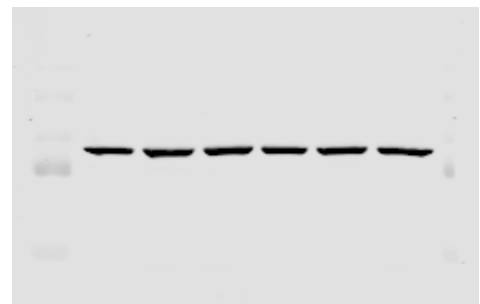

HSP90

e.

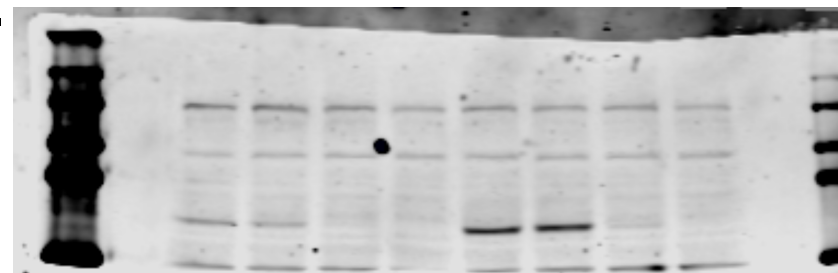

-P  
SREBP2  
-C

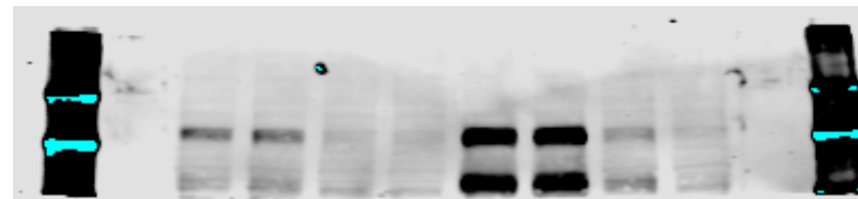

LDLR

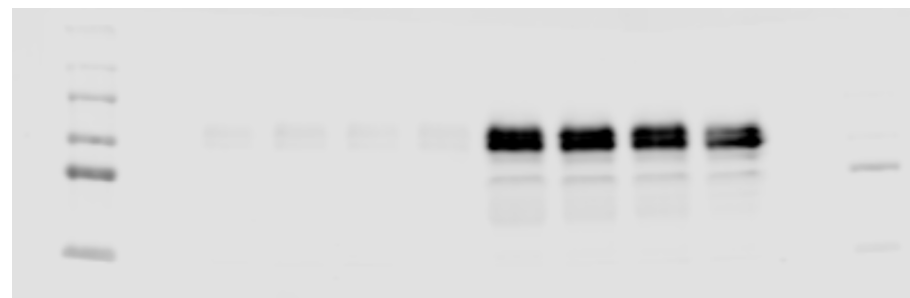

ICAM1

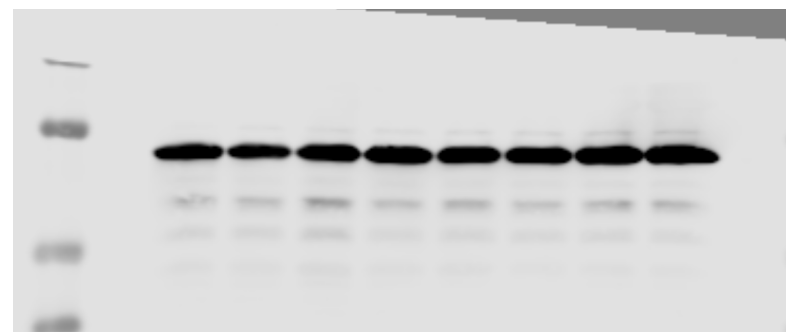

GAPDH

f.

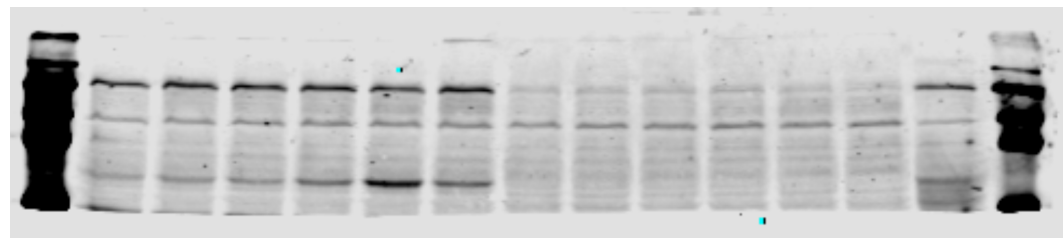

-P  
SREBP2  
-C

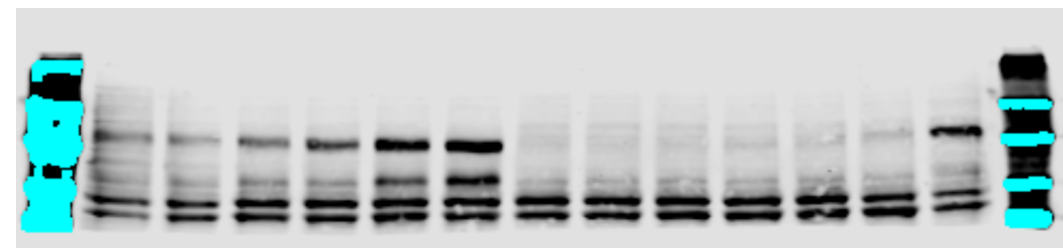

LDLR

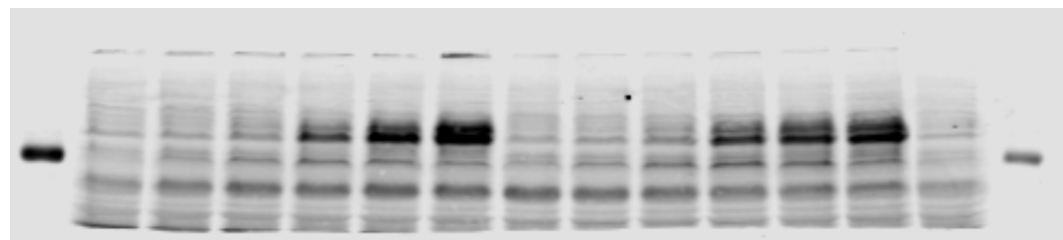

ICAM1

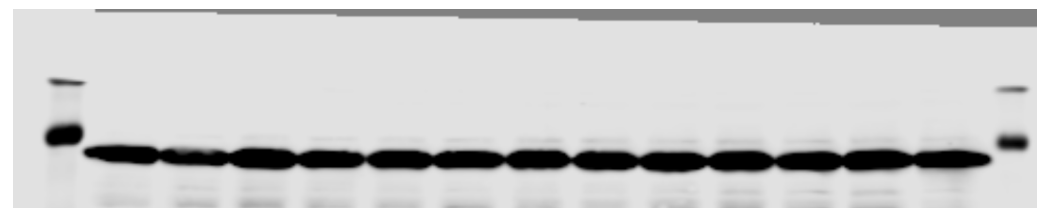

GAPDH

Supplement: Figure 4—source data 1. [file elife-79529-fig4-data1.pdf]

**a.**

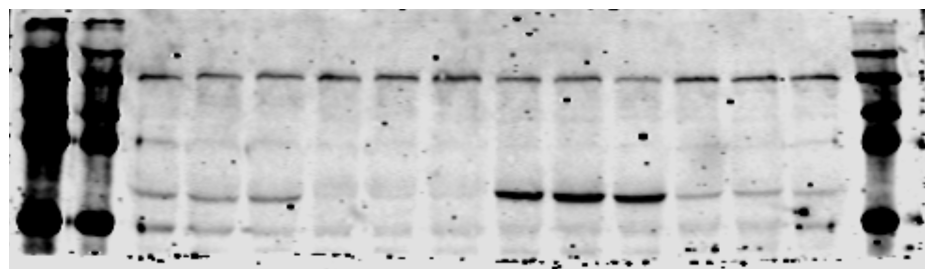

-P  
SREBP2  
-C

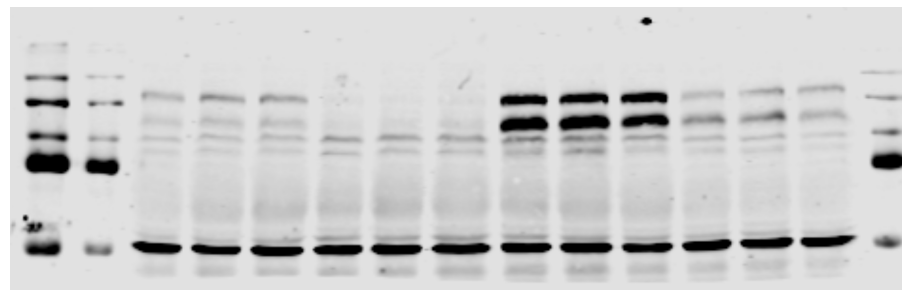

LDLR

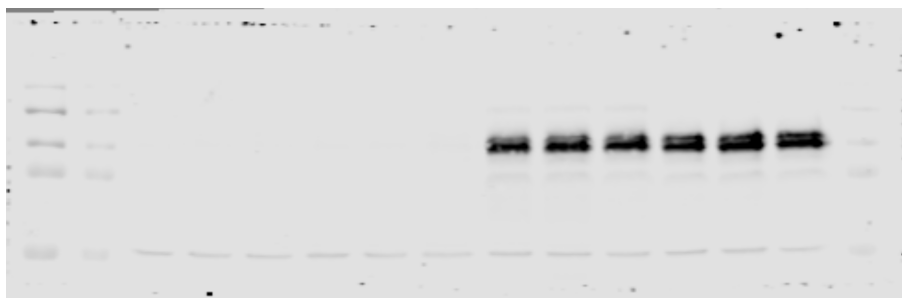

ICAM1

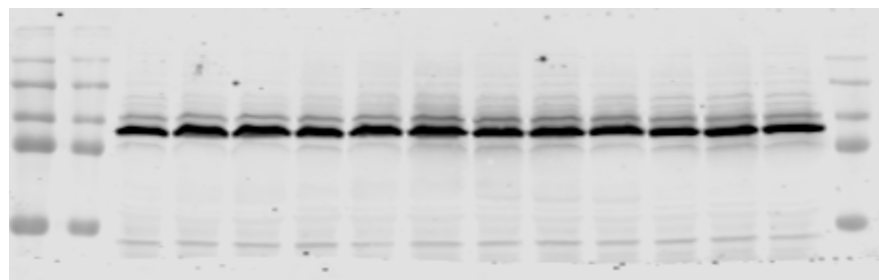

HSP90

**b.**

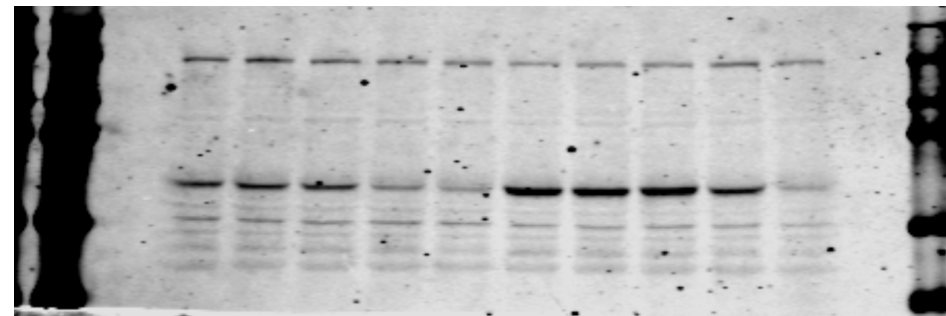

-P  
SREBP2  
-C

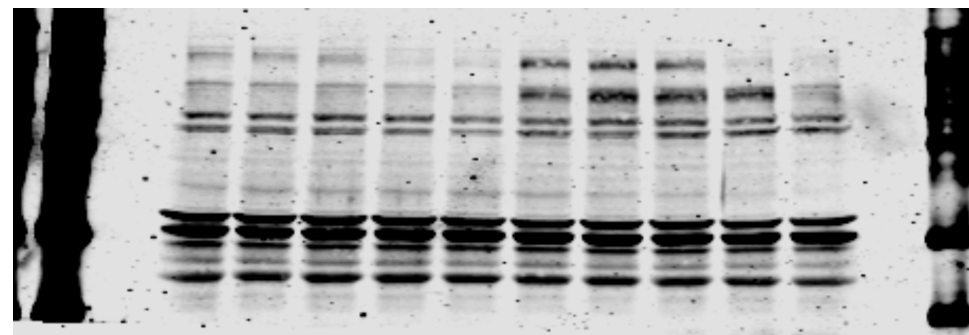

LDLR

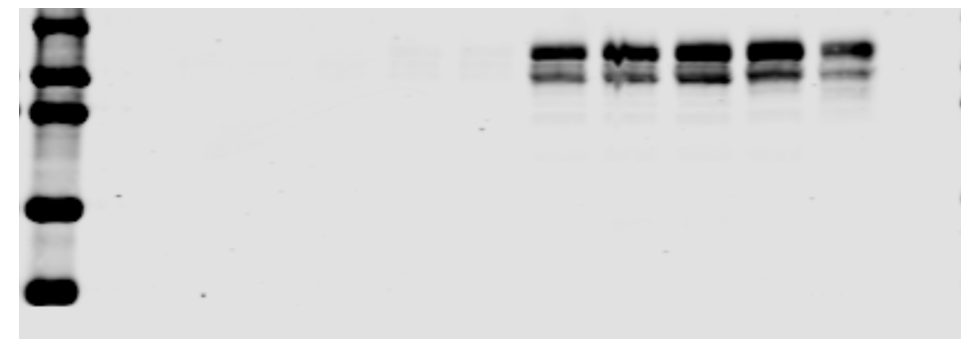

ICAM1

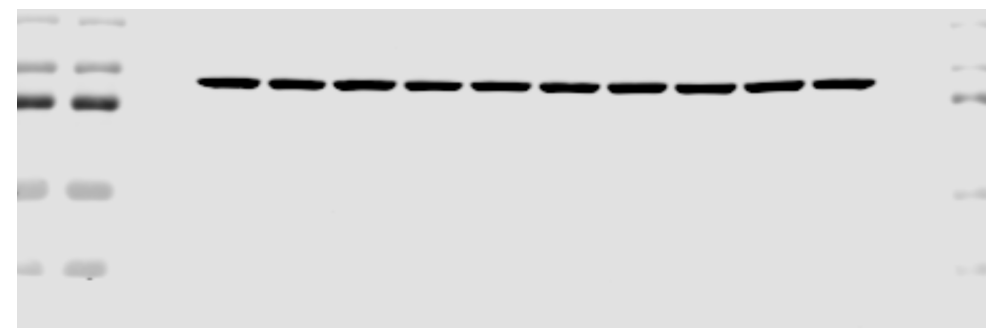

HSP90

c.

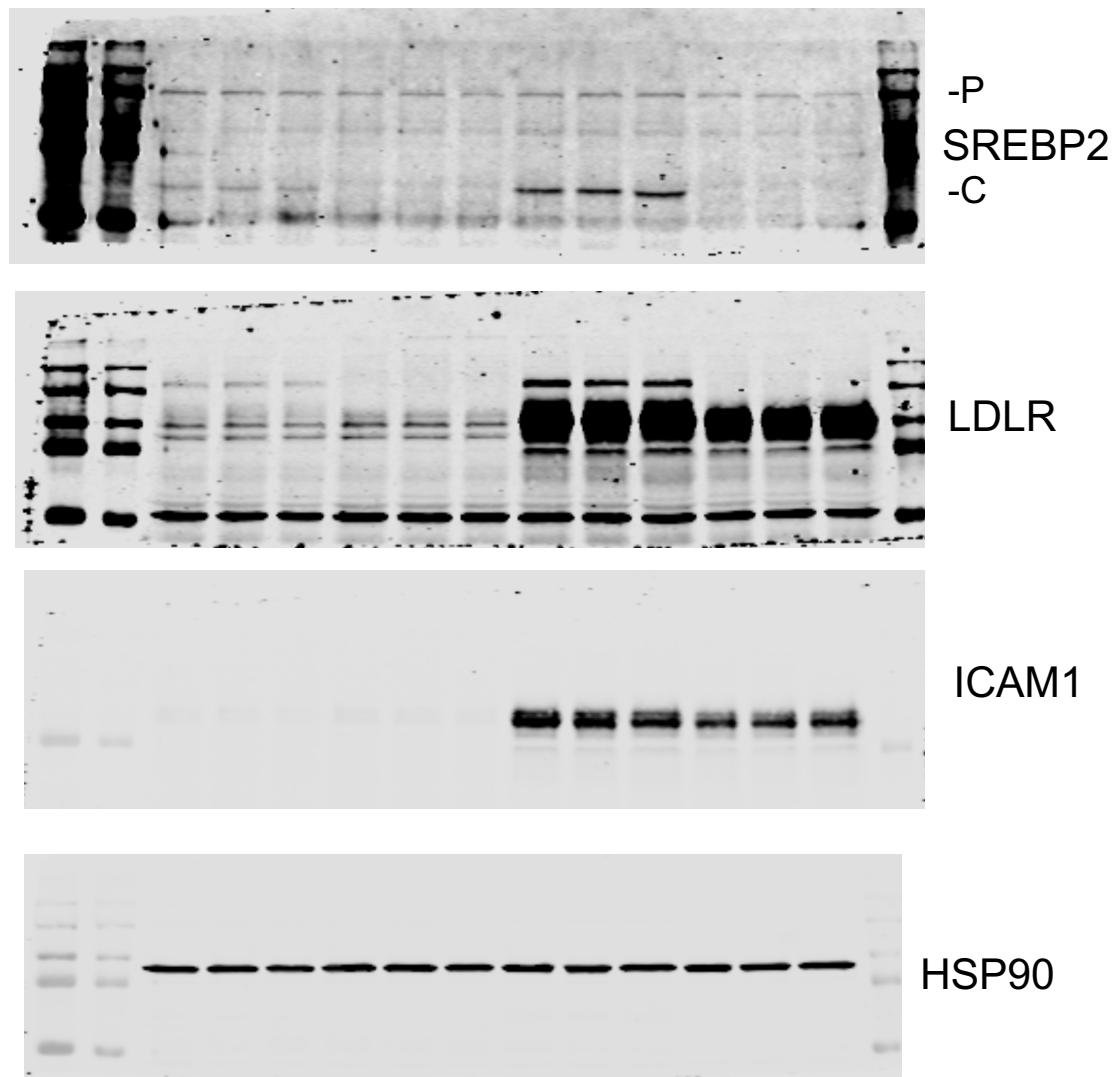

d.

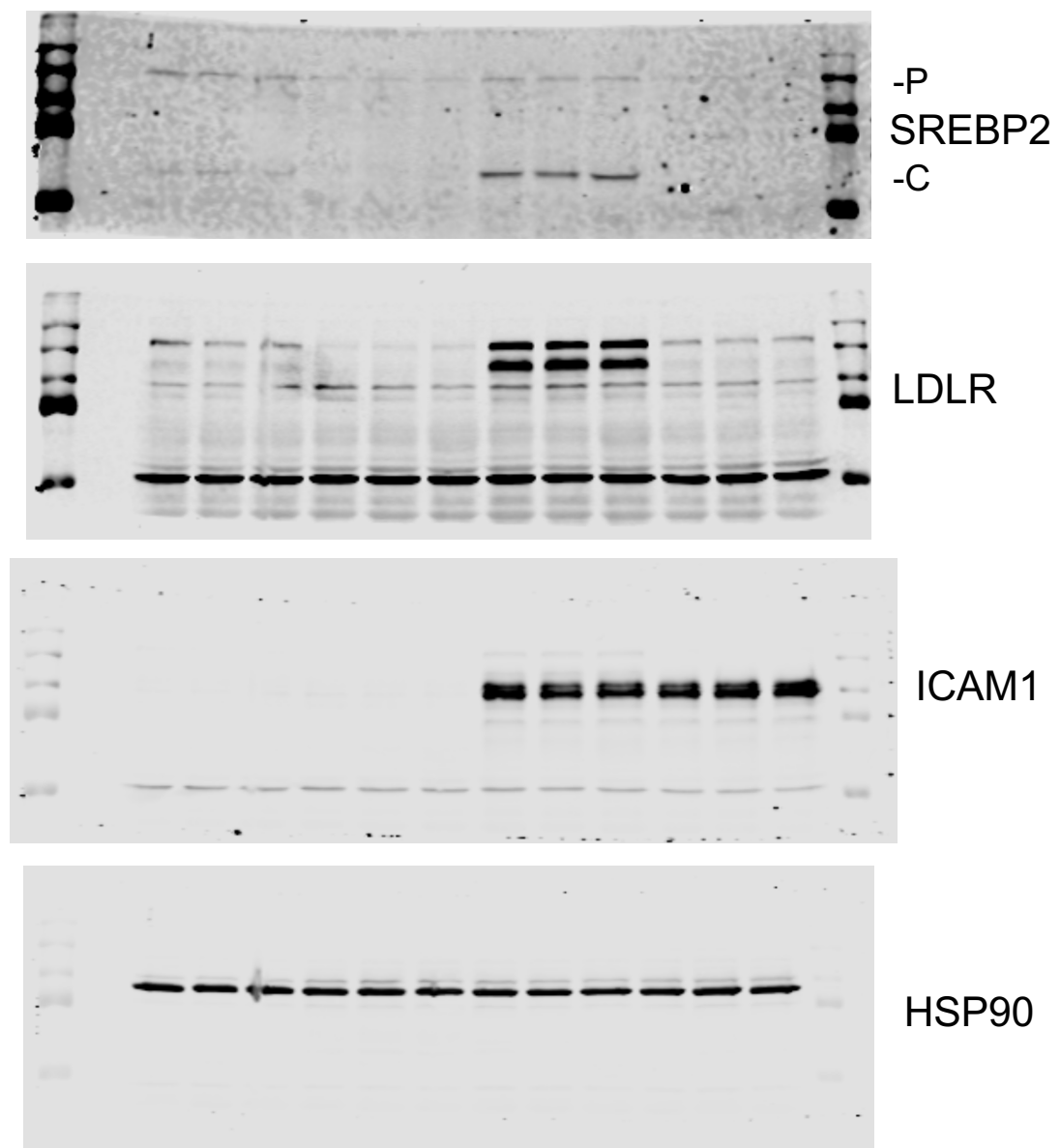

Supplement: Figure 4—figure supplement 1—source data 1. [file elife-79529-fig4-figsupp1-data1.pdf]

c.

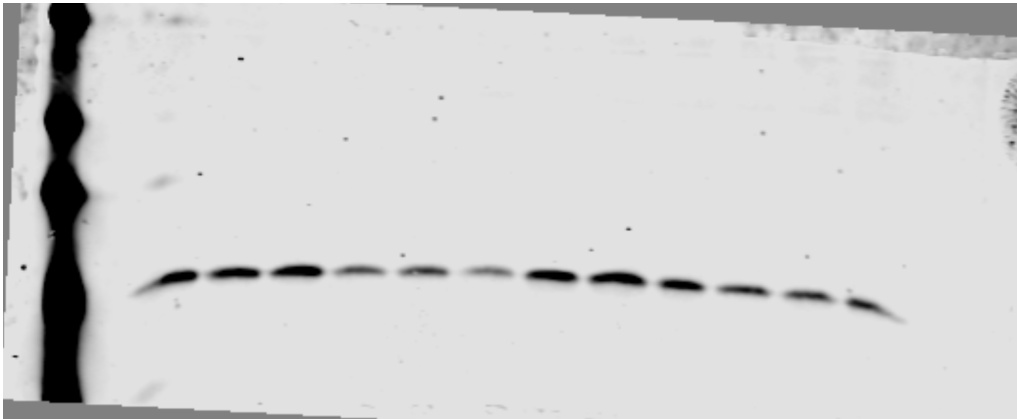

HIS (ALOD4)

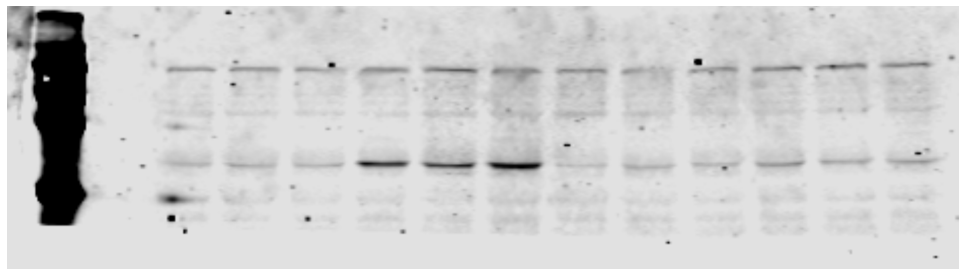

-P  
SREBP2  
-C

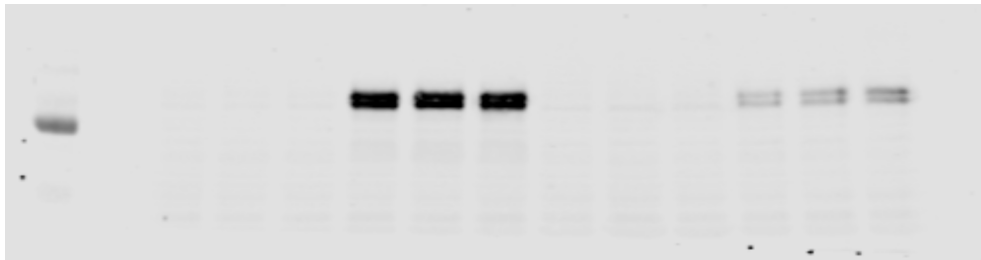

ICAM1

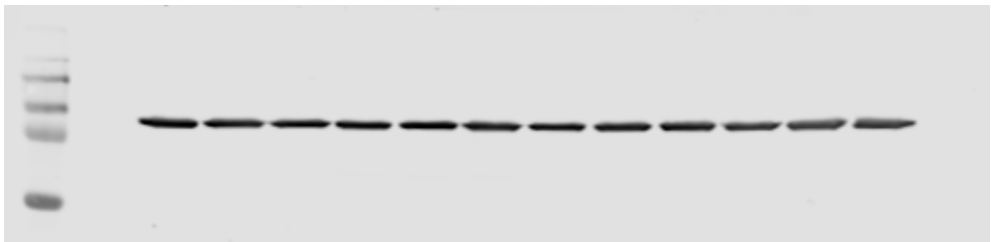

HSP90

f.

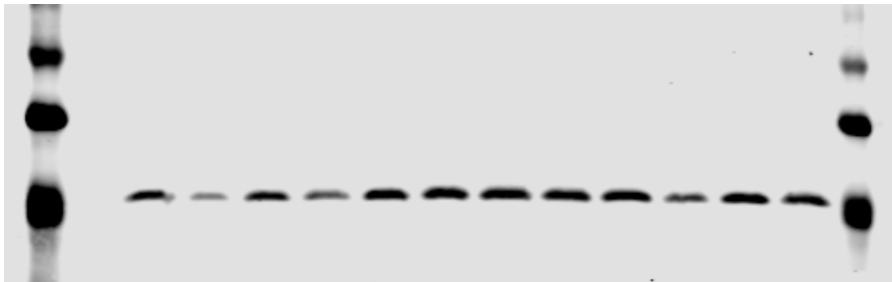

HIS  
(ALOD4)

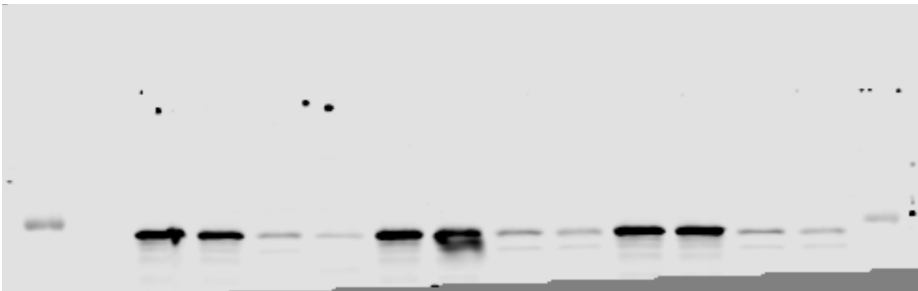

RELA

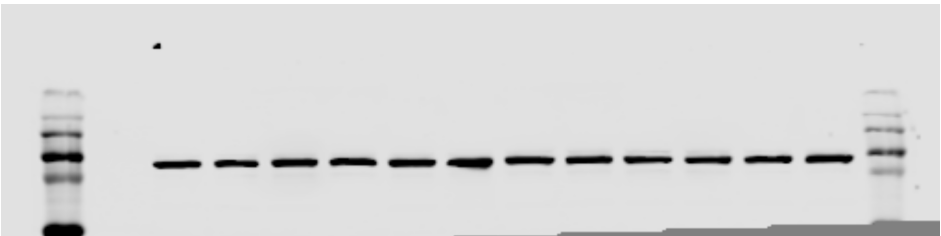

HSP90

Supplement: Figure 5—source data 1. [file elife-79529-fig5-data1.pdf]

a.

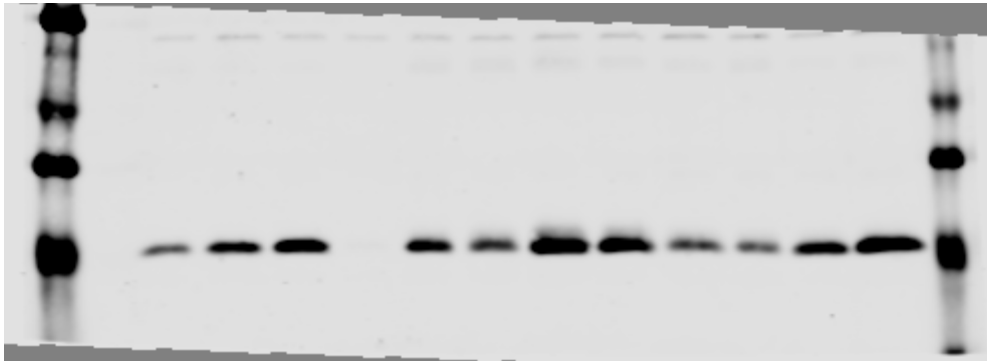

HIS  
(ALOD4)

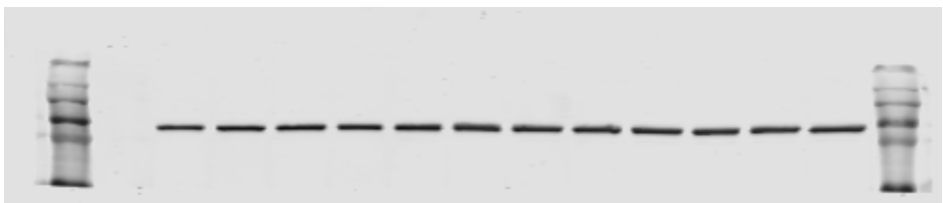

HSP90

c.

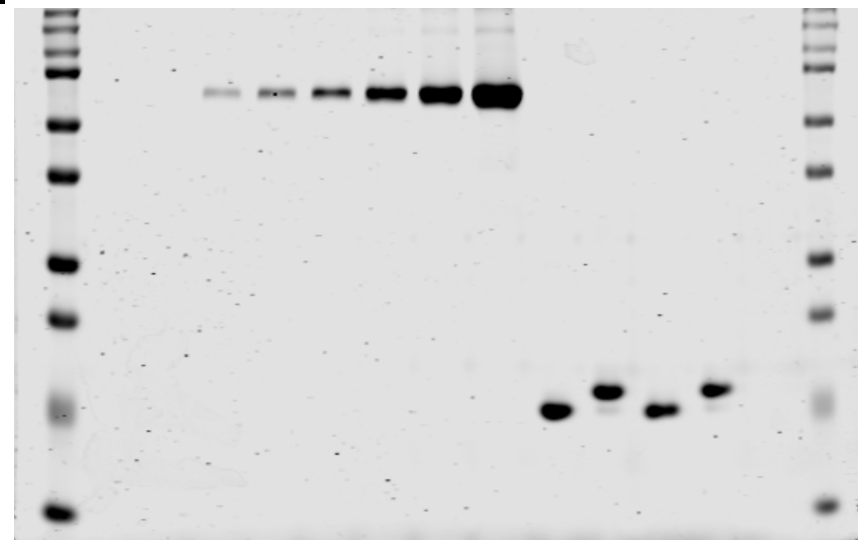

Coomassie

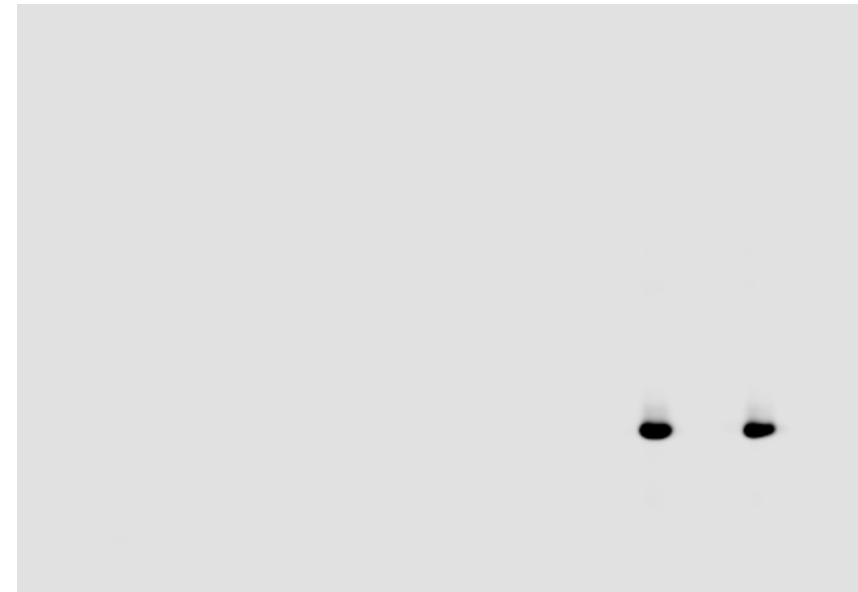

700

Supplement: Figure 5—figure supplement 1—source data 1. [file elife-79529-fig5-figsupp1-data1.pdf]

c.

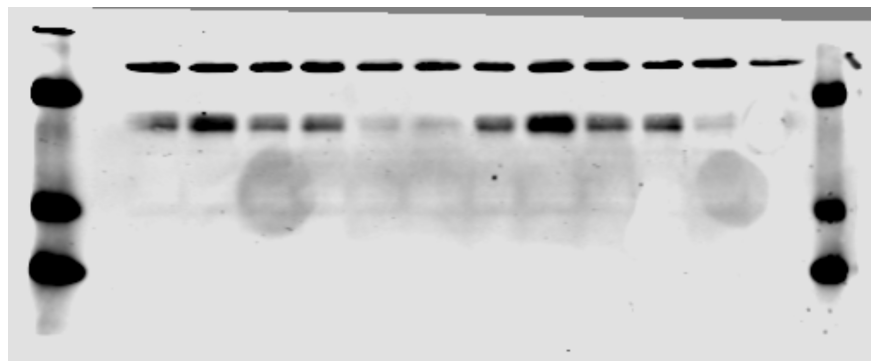

STARD10

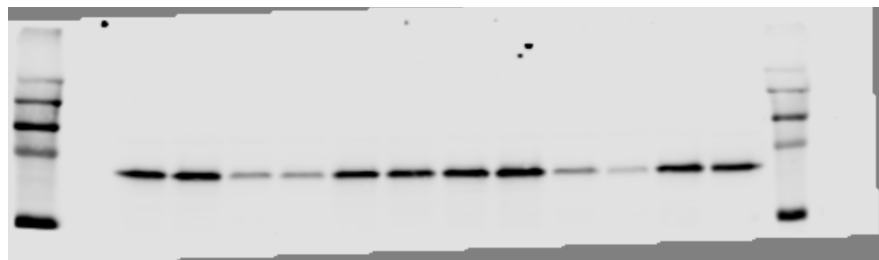

RELA

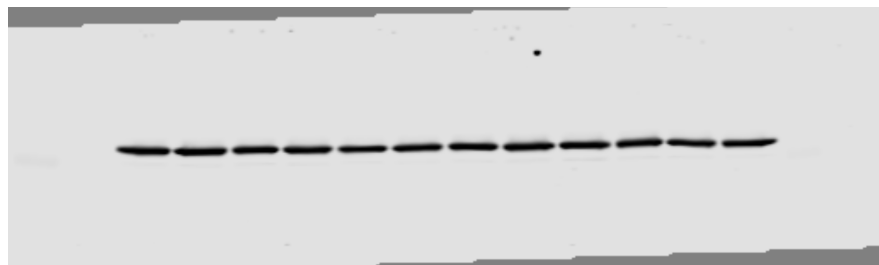

HSP90

e.

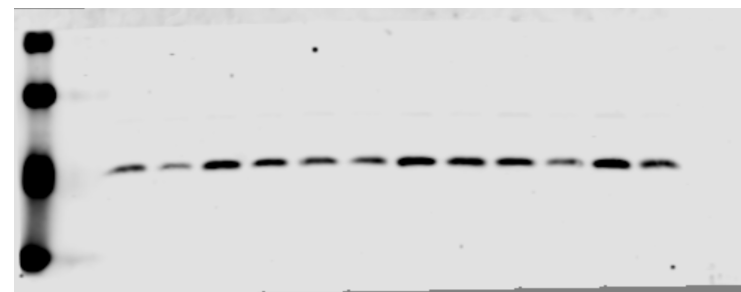

HIS (ALOD4)

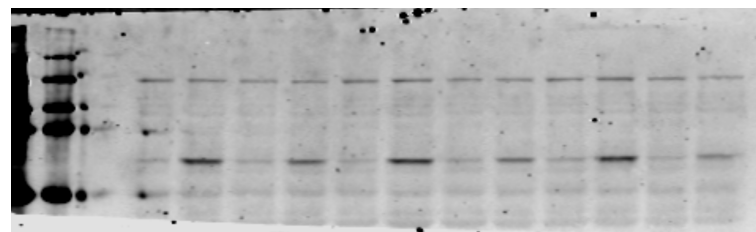

-P  
SREBP2  
-C

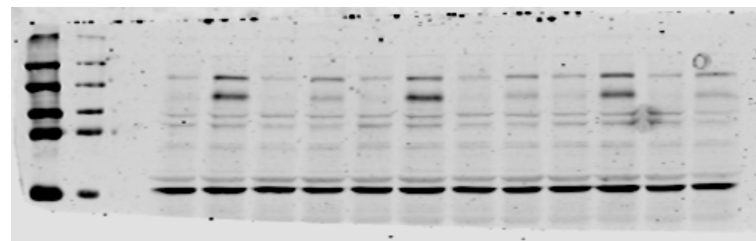

LDLR

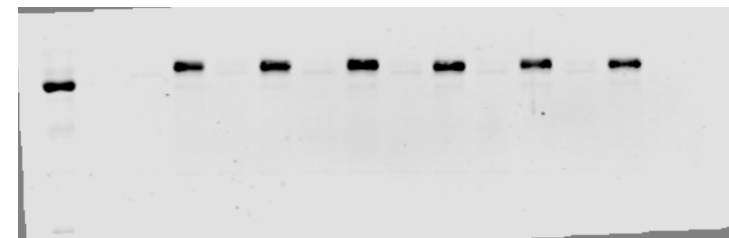

ICAM1

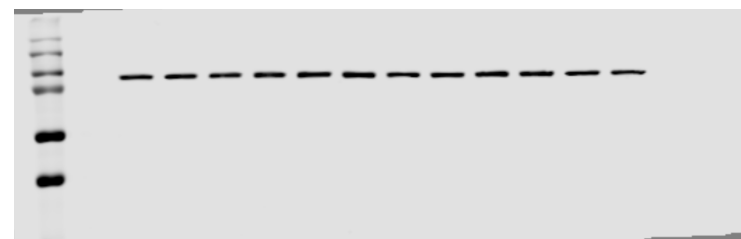

HSP90

Supplement: Figure 6—source data 1. [file elife-79529-fig6-data1.pdf]

c.

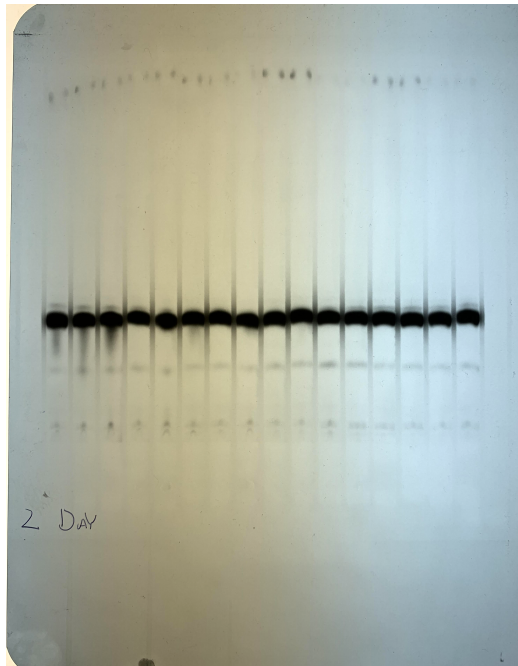

d.

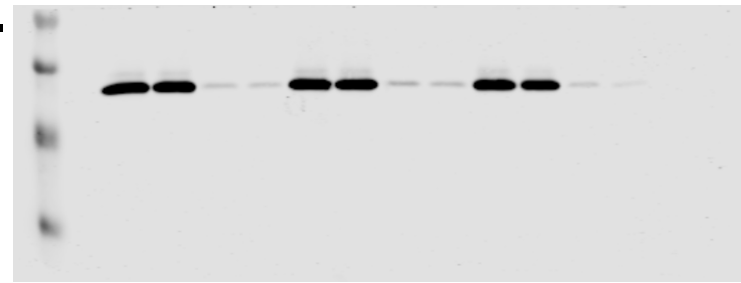

HIS  
(OlyA)

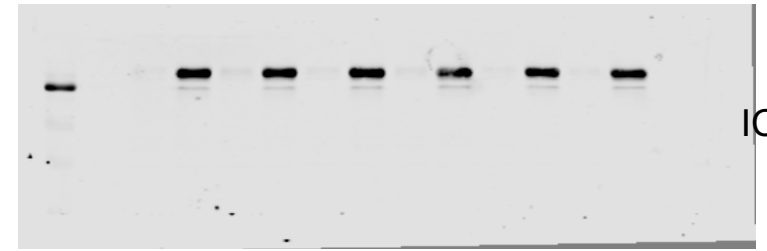

ICAM1

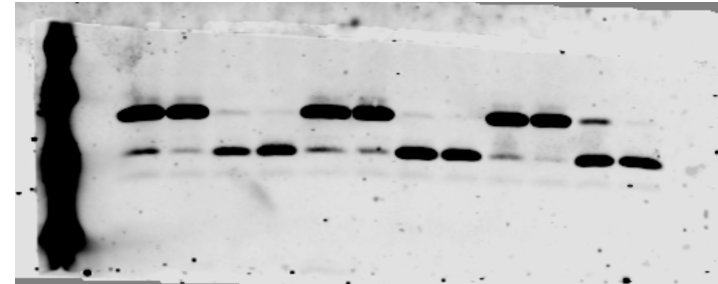

HIS  
(ALOD4)

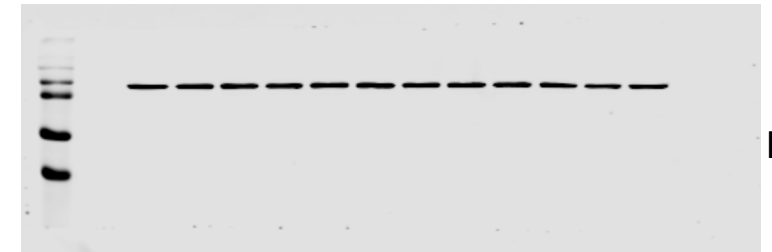

HSP90

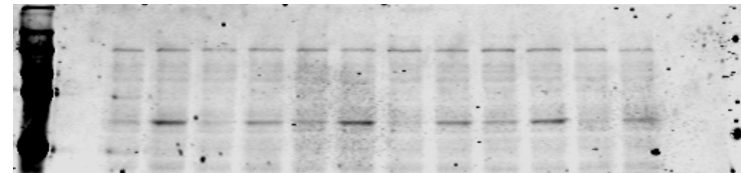

-P  
SREBP2  
-C

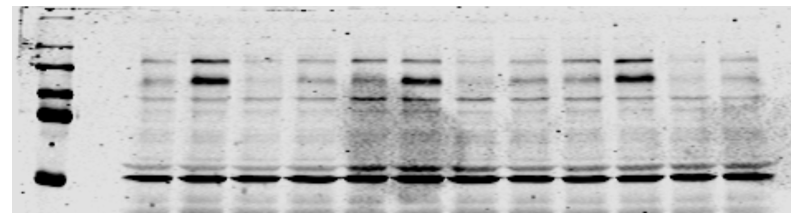

LDLR

f.

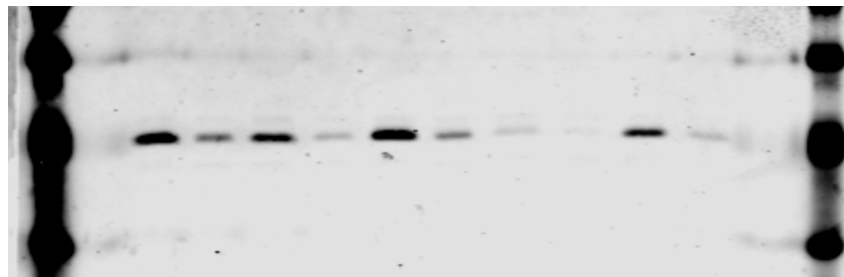

HIS (ALOD4)

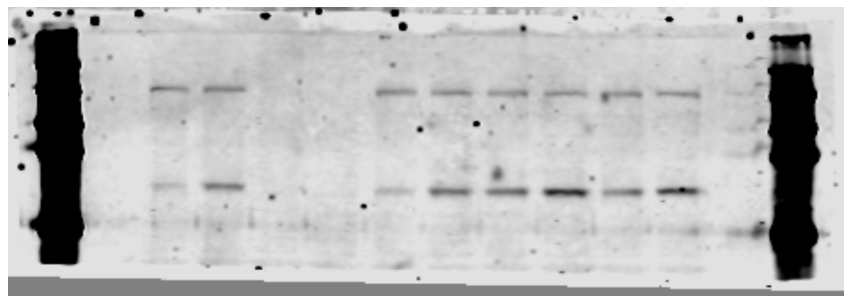

-P  
SREBP2  
-C

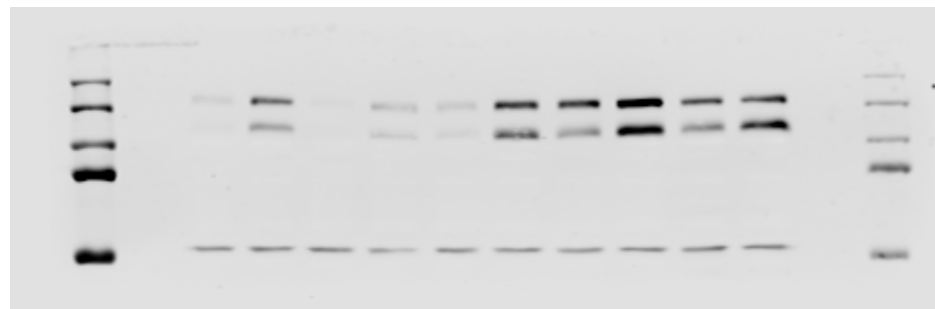

LDLR

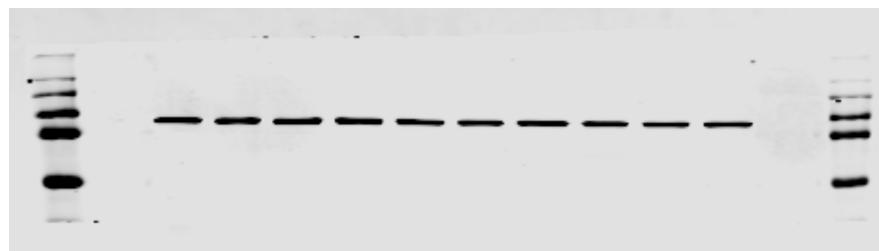

HSP90

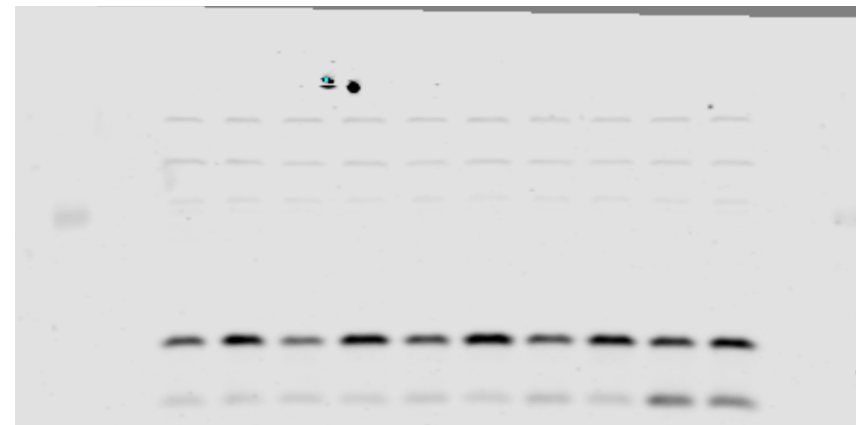

LC3b

Supplement: Figure 6—figure supplement 1—source data 1. [file elife-79529-fig6-figsupp1-data1.pdf]

**Figure 6 – Figure Supplement 3.**

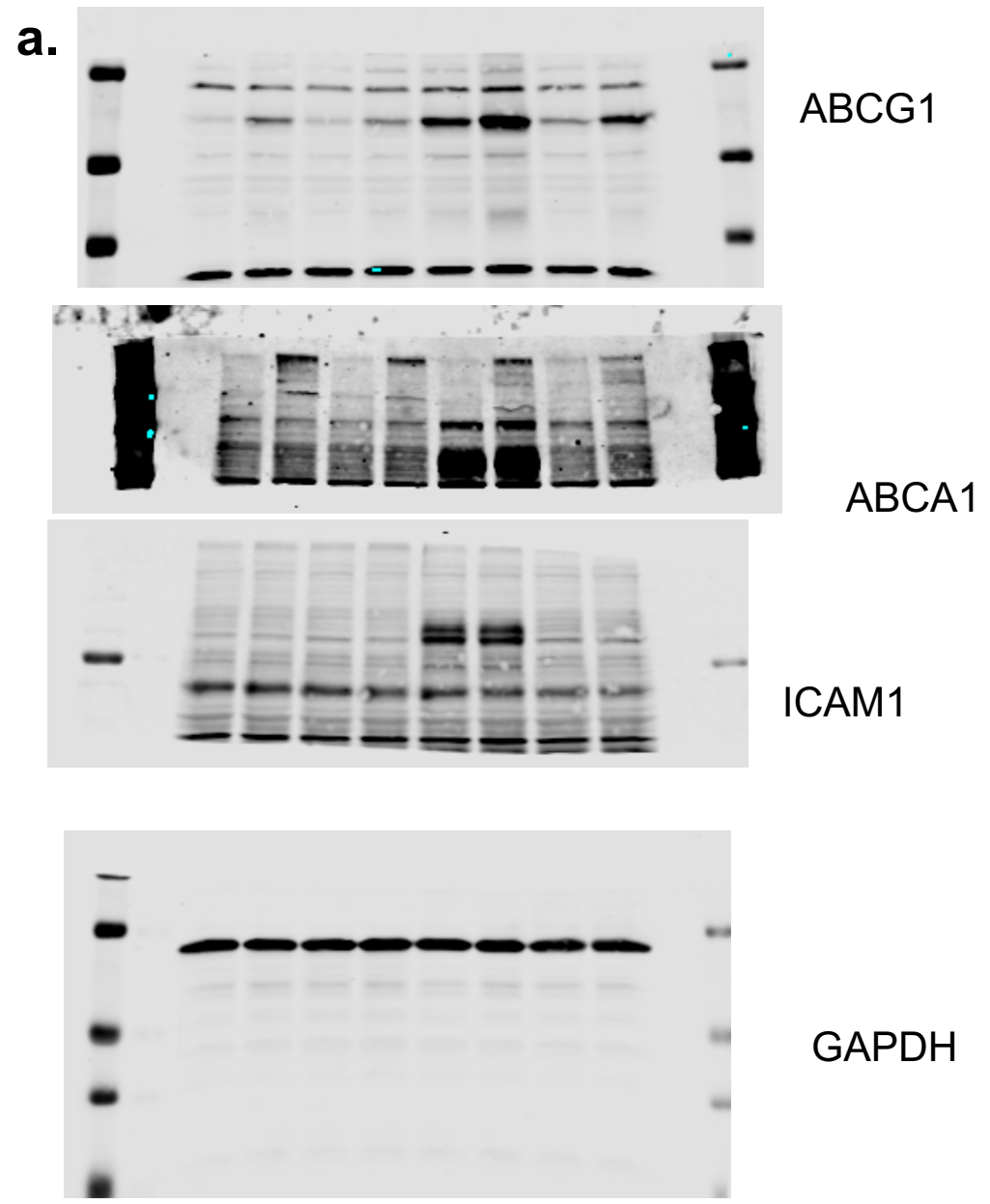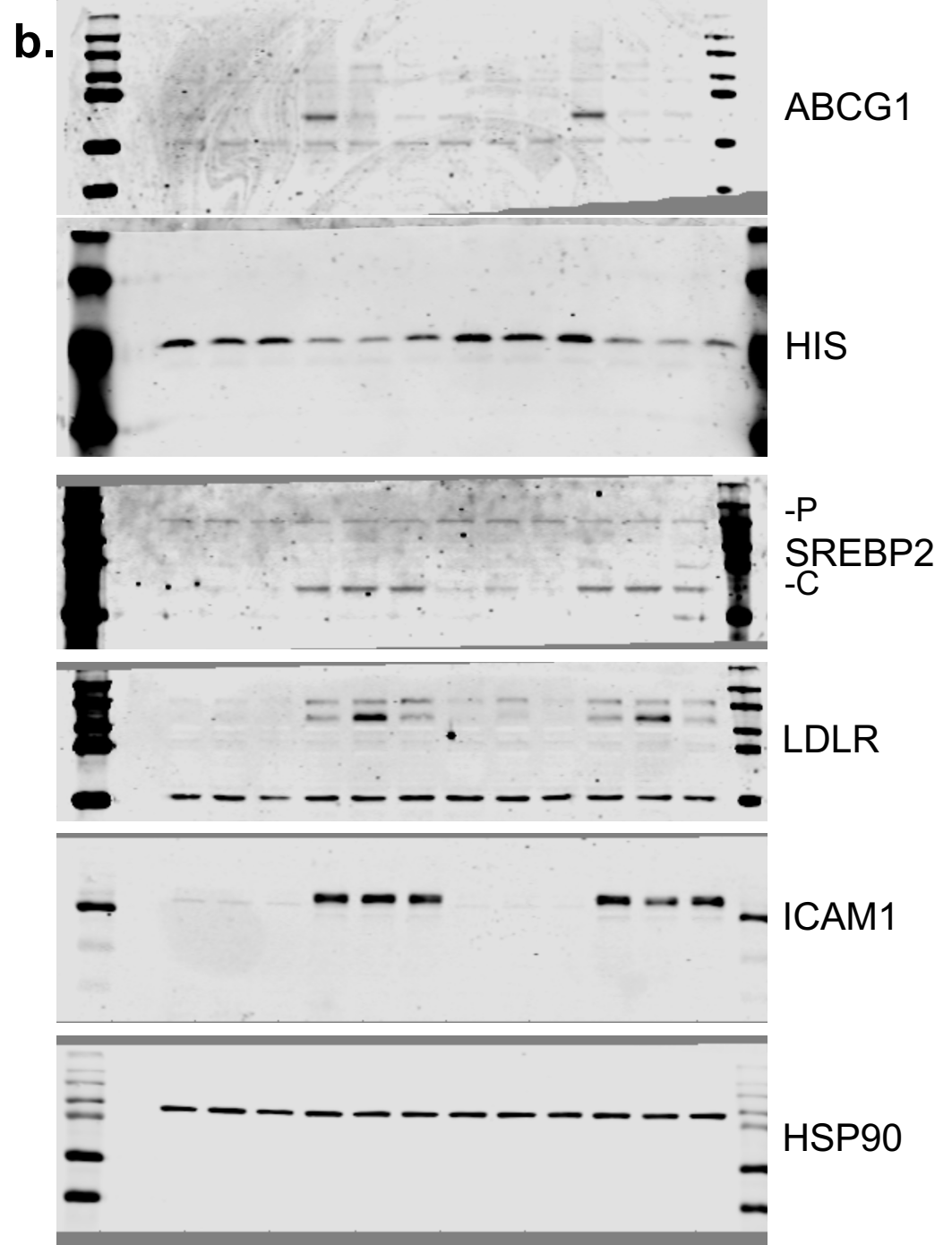

Supplement: Figure 6—figure supplement 3—source data 1. [file elife-79529-fig6-figsupp3-data1.pdf]
