# Supplementary material for: Inflammatory stress signaling via NF-kB alters accessible cholesterol to upregulate SREBP2 transcriptional activity in endothelial cells
Source: eLife. 2022 Aug 12;11:e79529. doi: 10.7554/eLife.79529 (PMC9395194; doi:10.7554/eLife.79529)

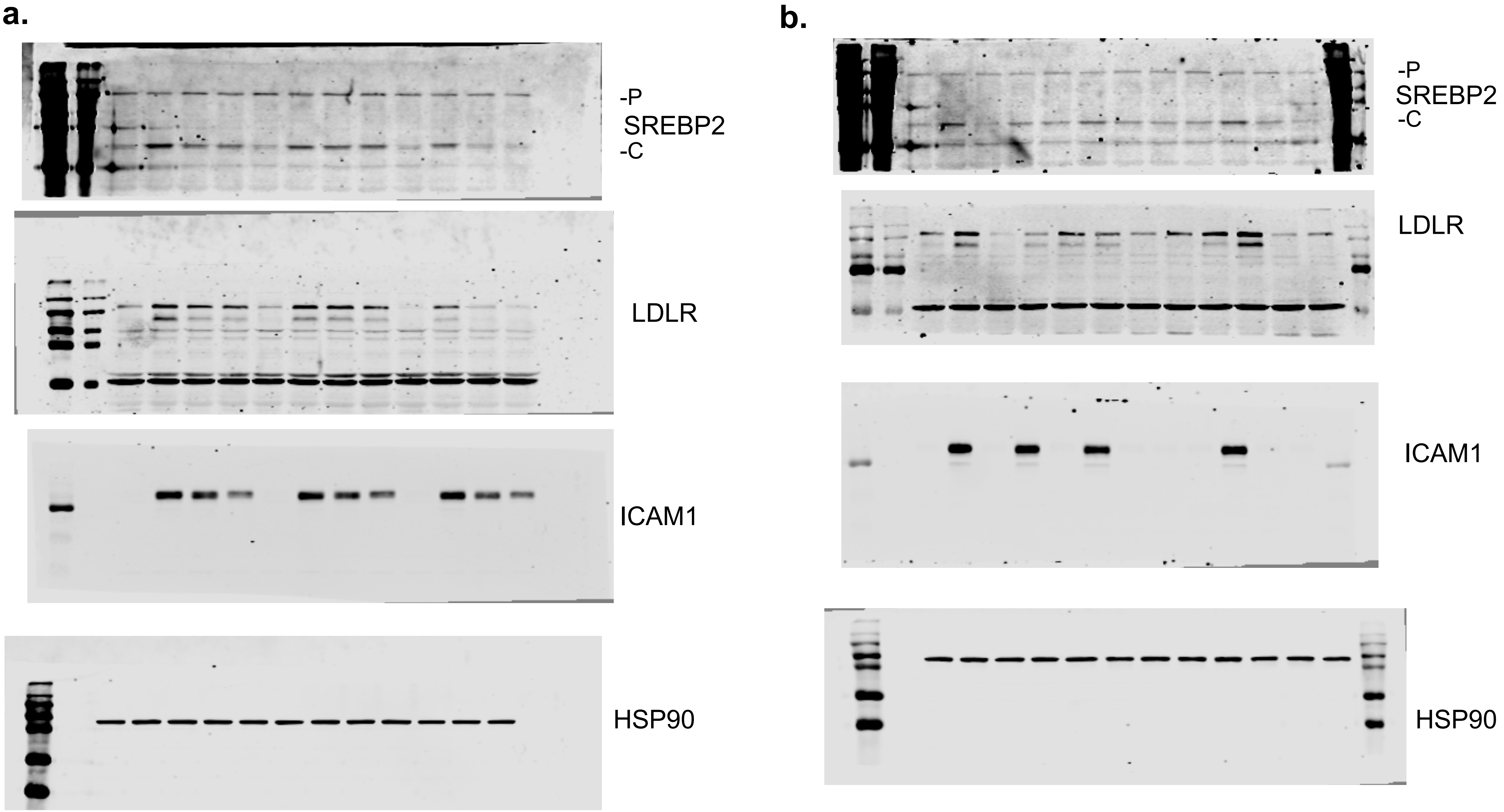
a.
-P
SREBP2
-C
LDLR
ICAM1
HSP90
b.
-P
SREBP2
-C
LDLR
ICAM1
HSP90

c.

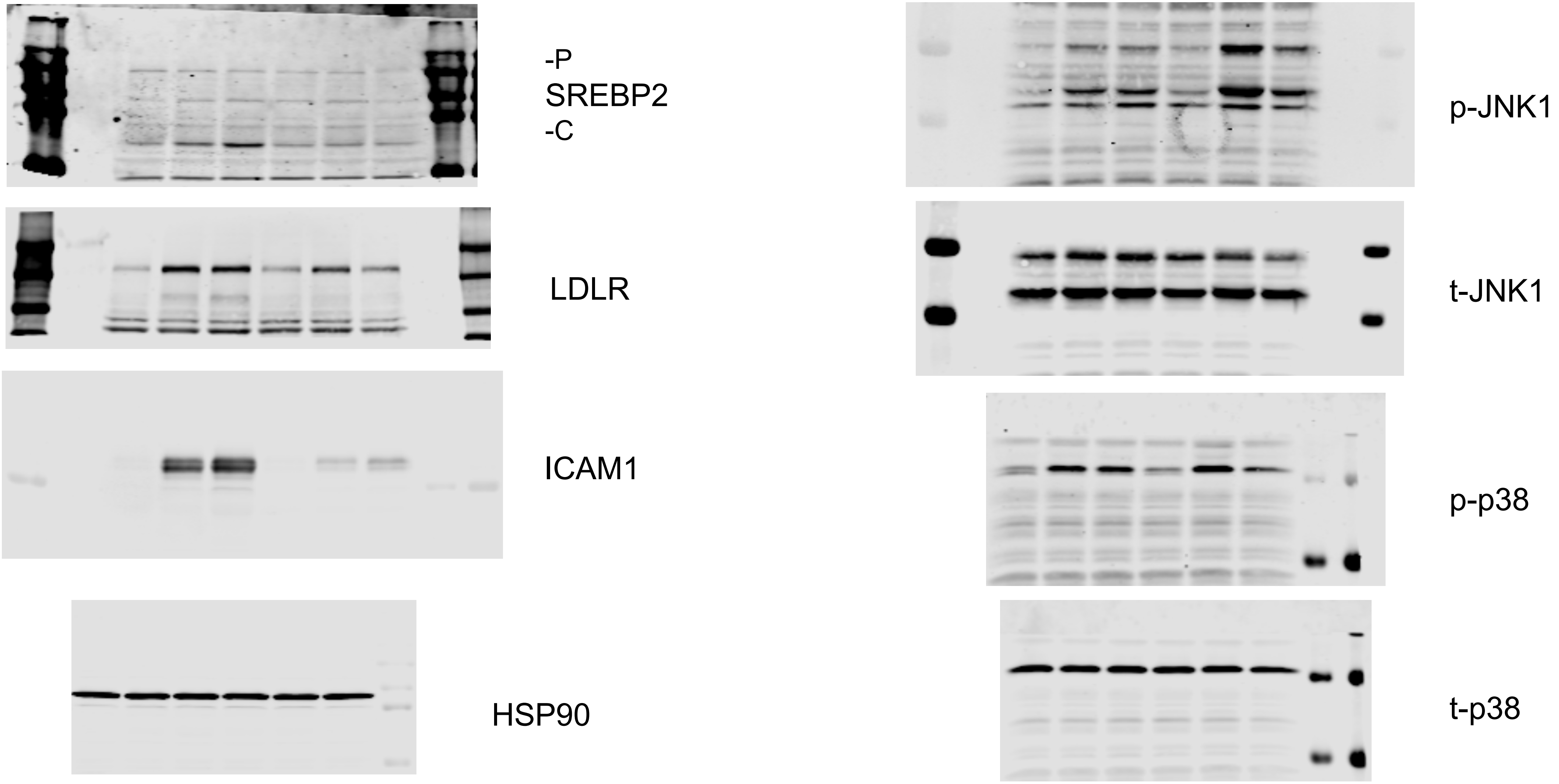

e.

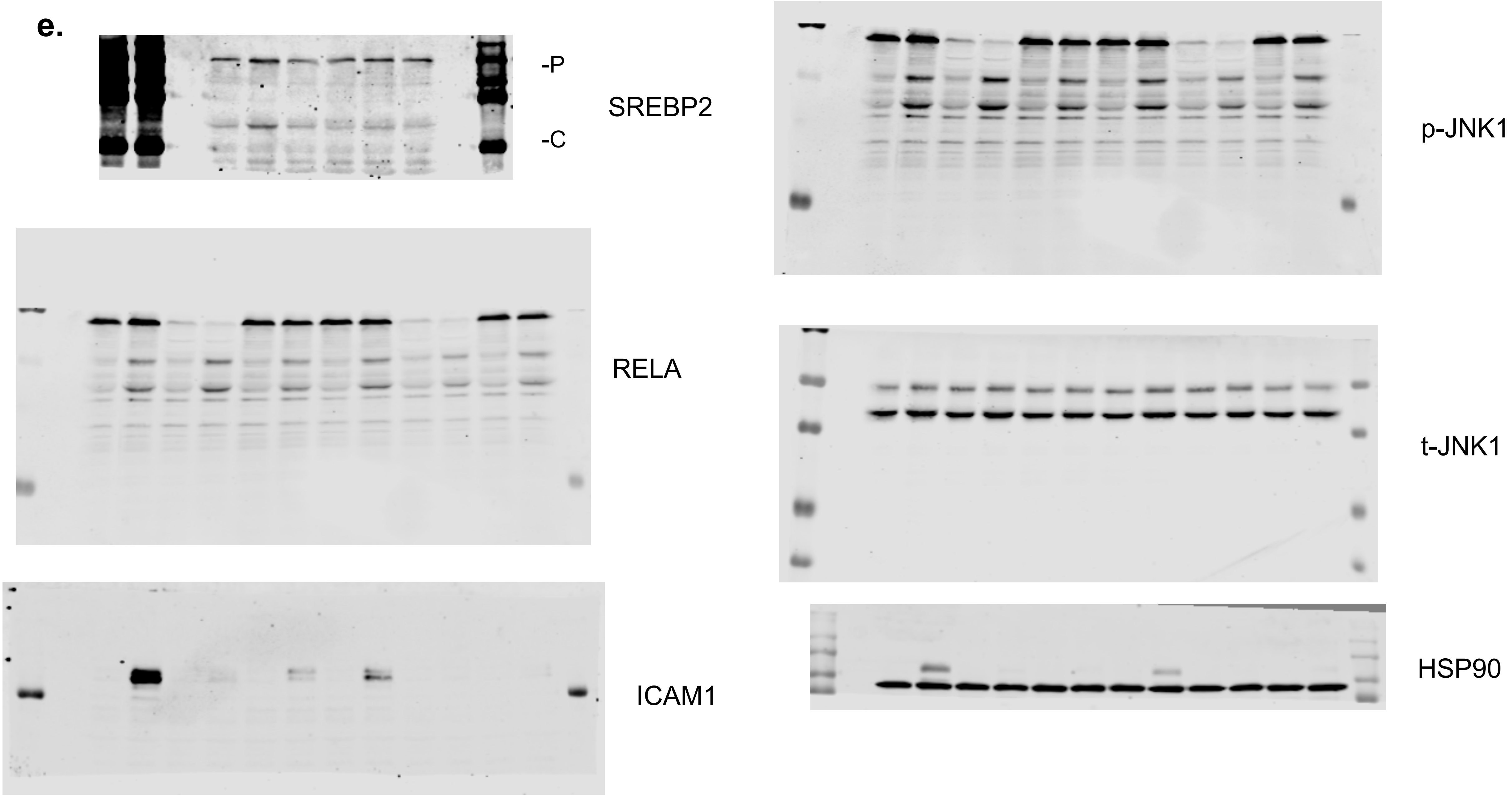

Supplement: Figure 3—source data 1. [file elife-79529-fig3-data1.pdf]
